# Glaucoma drainage devices in children: an updated review

**DOI:** 10.5935/0004-2749.2021-0338

**Published:** 2024-03-20

**Authors:** Alex Teles Vasconcelos, José Aloisio Massote, Cassia Senger, Lívia Prudente Barbieri, Sebastião Cronemberger, Jayter Silva Paula

**Affiliations:** 1 Department of Ophthalmology, Otorhinolaryngology, and Head and Neck Surgery, Faculdade de Medicina de Ribeirão Preto, Universidade de São Paulo, Ribeirão Preto, SP, Brazil.; 2 Department of Ophthalmology and Otorhinolaryngology, Universidade Federal de Minas Gerais, Belo Horizonte, MG, Brazil.; 3 Department of Pediatric Dentistry, Orthodontics and Public Health, Faculdade de Medicina de Bauru, Universidade de São Paulo, Bauru, SP, Brazil.; 4 Department of Surgery, Universidade Federal de Uberlândia, Uberlândia, MG, Brazil.

**Keywords:** Congenital glaucoma, Glaucoma drainage implants, Tonometry, ocular, Drainage, Intraocular pressure, Glaucoma congênito, Implantes para drenagem de glaucoma, Tonometria ocular, Drenagem, Pressão intraocular

## Abstract

Implantation of glaucoma drainage devices is a valuable therapeutic option, particularly in children with glaucoma refractory to primary surgical treatment. Glaucoma drainage devices are typically used when conjunctival scarring hampers filtration surgery or prior angle procedures are not effective in controlling intraocular pressure. Despite known complications, the use of glaucoma drainage devices in children has increased in recent years, even as the primary surgical option. In this review, we evaluate the results of recent studies involving the implantation of glaucoma drainage devices in children, discussing new advances, and comparing the success rates and complications of different devices.

## Abstract (pt)

O implante de dispositivos de drenagem para glaucoma (DDGs) é uma opção terapêutica valiosa, principalmente em crianças com glaucoma refratário ao tratamento cirúrgico primário. Os dispositivos de drenagem para glaucoma têm sido utilizados principalmente quando a cicatrização conjuntival dificulta a cirurgia fistulizante ou procedimentos angulares prévios não foram eficazes no controle da pressão intraocular. Apesar das complicações conhecidas, o uso de dispositivos de drenagem para glaucoma em crianças tem aumentado nos últimos anos, inclusive como opção cirúrgica primária. Nesta revisão, atualizamos os resultados de estudos recentes envolvendo o implante de dispositivos de drenagem para glaucoma em crianças, discutindo novos avanços e comparando diferentes dispositivos, taxas de sucesso e complicações.

## INTRODUCTION

Childhood glaucoma is a rare and heterogeneous group of ocular disorders that requires meticulous care and specialized management to prevent permanent vision loss. The condition is characterized by elevated intraocular pressure (IOP)-related damage to the optic nerve^(1)^.

In 2013, the Childhood Glaucoma Research Network developed the first international consensus classification for childhood glaucoma^(1)^. In this consensus which includes glaucoma and suspected glaucoma, childhood glaucoma is classified as primary and secondary. There are two types of primary childhood glaucoma (unknown cause): primary congenital glaucoma (PCG) and juvenile open-angle glaucoma (JOAG). All other types of glaucoma in children are referred to as secondary glaucoma (SG)^(1)^.

Primary congenital glaucoma (PCG) is a developmental glaucoma occurring before the age of three years (roughly 90% manifesting in the first days or months of life) due to an obstruction that prevents adequate drainage of aqueous humor caused by abnormal development of the trabecular meshwork. It is a rare disease with variable incidence across countries and ethnic groups. Its incidence in western countries (Ireland, Britain, and the USA) lies within 1 to 10 per 20,000 live births^(2,3,4,5,6)^. The incidence of PCG is higher in the Middle East, including Saudi Arabia, where consanguinity in marriages are more prevalent and the estimated incidence is 1 per 2,500 live births^(7,8)^. In Brazil, there are no accurate data on either the incidence or prevalence of glaucoma in childhood.

The main treatment goal in childhood glaucoma is to maintain vision for lifelong by controlling the IOP^(1)^. Therefore, treatment of childhood glaucoma is typically challenging, especially in children with other complex ocular dysgenesis such as Peters’ anomaly, aniridia, Axenfeld-Rieger Syndrome, and Sturge-Weber Syndrome.

Traditionally, a stepwise “staged approach” is used for surgical decision-making^(9)^. The initial management usually involves angle surgeries, mostly goniotomy or trabeculotomy (TROC)^(10)^. Goniotomy is often the procedure of choice since it entails preservation of conjunctiva; however, TROC can be performed in swollen or opaque corneas achieving better success rates^(10)^. Both procedures may be repeated in case of failure and are associated with good success rates; however, the overall failure rate is 20% and many children require additional surgery to control IOP in the long term^(10)^. In this event, the next steps in such cases include a combined trabeculotomy-trabeculectomy^(11)^ or trabeculectomy (TRAB) with mitomycin-C (MMC)^(12)^ and, lastly, implantation of a glaucoma drainage device (GDD)^(13,14,15,16,17)^ or cyclodestruction of ciliary body for very advanced glaucoma^(18)^. Cyclodestructive procedures have limited long-term success in children and can sometimes lead to vision loss^(18)^.

Different GDDs have been used to treat refractory glaucomas or as a primary procedure in children in whom the outcomes of TRAB are expected to be poor^(19)^. Currently, there is a paucity of data regarding the longterm outcomes of GDDs implanted for the treatment of pediatric glaucoma. This is attributable to several factors including difficulties in performing studies of such rare and complex diseases, lack of reliable data for comparing several ocular conditions and outcomes in children, as well as insufficient follow-up time^(15,16,17)^. In addition, most studies are retrospective because of the ethical issues.

A previous systematic review of aqueous shunt in glaucoma has summarized many of these clinical aspects, but it mostly pertained to adult patients^(19)^. Moreover, a direct comparison between surgical results of GDD in children and adults is not meaningful. Children typically require general anesthesia and present variable ocular malformations, more elastic sclera, and different healing processes^(9,12)^.

Nassiri et al. (2011) reviewed the use of Ahmed glaucoma valve (AGV) in children^(15)^. Since then, no studies have reviewed the use of GDD implants in pediatric glaucoma. The purpose of this narrative review was to summarize the available information regarding the outcomes of GDD implantation in children considering the recent advances in GDD surgical techniques, new devices, and postoperative management.

## METHODS

### Literature selection

A literature search was relevant studies was conducted on biomedical databases (MEDLINE, Google Scholar, Ovid, Cochrane^(19)^, and EMBASE) covering the period from January 2006 to June 2022.

The following keywords were used for the literature search: “glaucoma” and “pediatric” and “drainage implant” OR “glaucoma” and “pediatric” and “drainage device” OR “glaucoma” and “childhood” and “drainage implant” OR “glaucoma” and “childhood” and “drainage device” OR “glaucoma” and “congenital” and “drainage implant” OR “glaucoma” and “congenital” and “drainage device”.

### Inclusion criteria

1) Original research articles published between January 2006 to June 2022; 2) language of publication: English; 3) studies published in high-impact journals based on SC Imago Journal Rank indicator (Q1 and Q2 journals).

### Exclusion criteria

1) Case reports and abstracts; 2) main outcome measure not related to GDD implantation in children; 3) lack of availability of complete results.

### Data collection

Data pertaining to the following variables were compiled: age of children at the time of GDD implantation; number of eyes operated; initial and final IOP, success rate, follow-up period (months), and complications. All the authors of this review participated in the literature selection as well as in the analysis and redaction of the work.

Descriptive analyses were performed considering the GDD type (valved or nonvalved), surgical technique, indications, clinical outcomes, and complications.

## RESULTS

We reviewed 58 studies that qualified our criteria; of these, 23 studies that compared specific differences among GDD in children are presented in table 1.

**Table 1:** Fifteen-years update on the use of glaucoma drainage device in pediatric glaucoma

Studies	Devices	Indication	Age* (Yrs.)	# Eyes	Initial IOP* (mmHg)	Final IOP* (mmHg)	% Success rate (Ys)	Mean follow-up (mos.)	Complications (%)
van Overdam et al., 2006^(20)^	BGI	PCG and SCG	3.3 ± 3.6	55	27.2 ± 7.9	15.3 ± 6.6	94 (2) 85 (3)	31.9	Tube dislocation (14.5); tube exposure (5.5); cataract (5.5); phthisis (3.7); visual loss (7.3)
Al-Mobarak et al., 2009^(21)^	AGV	PCG and SCG	1.0 ± 0.5	42	33.5 ± 8.6	20.0 ± 9.7	73.8 (1) 63.3 (2)	24	Tube malpositioning requiring intervention (26.2); endophthalmitis (7.1); retinal detachment (7.1)
Al-Mobarak et al., 2009^(22)^	AGVp w/MMC AGVp w/o MMC	PCG and SCG	1.0 ± 0.5 0.9 ± 0.4	16/31 15/31	35.0 ± 11.4 31.6 ± 7.4	21.3 ± 13.1 18.9 ± 7.2	31.3 (2) 80.0 (2)	24	Tube migration requiring intervention (32.3); endophthalmitis (6.5); preseptal cellulitis (6.5); suprachoroidal hemorrhage (3.2)
Khan et al. 2009^(23)^	AGVs AGVp	PCG and SCG	1.2 ± 0.5 0.9 ± 0.5	11/42 31/42	33.9 ± 4.9 33.4 ± 9.7	19.8 ± 7.4 20.1 ± 10.5	90.9 (2) 54.8 (2)	24	Tube malpositioning requiring intervention (26.2); endophthalmitis (7.1); retinal detachment (7.1).
El Gendy et al., 2012^(24)^	BGI AGV	PCG and SCG	5.4 6.7	20 11	33.8 ± 5.7 39.8 ± 6.2	18 ± 5.7 24 ± 5.4	80 (4) 54.5 (2.7)	45.6 32.4	NA**
Tai & Song, 2014^(25)^	BGI	PCG and SCG	4.3 ± 4.8	45	31.6 ± 5.0	18.3 ± 4.8	86.7 (2)	24	Tube erosion (4.5); corneal decompensation (2.2); cataract (2.2); bleb encapsulation (2.2)
Razeghinejad et al., 2014^(26)^	AGV	Refractory PCG	2.7 ± 3.1	33	32.8 ± 7.3	16.8 ± 4.0	97.0 (1) 85.0 (2) 56.3 (5)	60	Tube-endothelial touch (9); tube extrusion (3); bleb encapsulation (**); cataract (6); retinal detachment
Chen et al., 2015^(13)^	AGV	PCG and SCG	6.8 ± 5.7	119	29.2 ± 9.7	15.2 ± 3.5	85.7 (1) 55.0 (5)	73.2	Tube exposure (7.6); tube touching the cornea (3.4); cataract (4.2); strabismus (2.5)
Mandalos et al., 2016^(17,27)^	BGI MGI	PCG and SCG	8.3 ± 5.1	53 16	29.4 ± 8.9	15.5 ± 8.4	66.7	45.7	Hypotony (52); choroidal effusion (27.5); bleb encapsulation (17.3); cataract (23); uveitis (20); decompensation (11.6); corneal touch (7.2)
Kaushik et al., 2017^(28)^	AADI	PCG and SCG	8.2 ± 3.6	34	27.4 ± 7.5	12.8 ± 5.6	81.7 (2)	18.3	Retinal detachment; tube malpositioning; tube-endothelial touch; conjunctival retraction; cataract.
Eksioglu et al., 2017^(29)^	AGV	SCG	14.2 ± 3.3	16	33.5 ± 7.3	12.7 ±3.2	49.2 (3)	64.4	Cataract (50.0); partial occlusion of the tube (12.5); hypotony (12.5); Descemet detachment (6.3); hyphema (6.3); retinal detachment (6.3).
Al-Haddad et al., 2018^(30)^	AGV	PCG and SCG	12.7 ± 9.3	11	24.8 ± 6.8	17.5 ± 4.3	82 (4.3)	51.6	NA**
Senthil et al., 2018^(31)^	AGV	PCG and SCG	3	76	29 ± 30	14.6 ± 15.4	88 (1)	27	16% overall**: hypotony; corneal touch; tube retraction; tube exposure.
Esfandiari et al., 2019 ^(32)^	AGV BGI	Pediatric GFCS	4.4 ± 2.1 4.1 ± 2.1	16 12	28.6 ± 4.1 30.0 ± 4.1	17.6 ± 2.1 17.6 ± 1.7	74.9 (4) 72.9 (4)	48	Hyphema (26.9); choroidal effusion (3.6); hypotony (3.6); tube exposure (7.2); corneal touch (7.2)
Pakravan et al., 2019^(16)^	AGV	PCG and SCG	7.9 ± 6.5	95	31.6 ± 8	16.4 ± 5.1	90 (1) 52.5 (5)	72	Tube elongation (8.4); leakage (2); tube exposure (2); endophthalmitis (1); anterior displacement (1); suprachoroidal hemorrhage (1); hypotony (1); Tenon cyst (1); effusion (1); AGV extrusion (1).
Rateb et al., 2019^(33)^	BGI AAID	PCG and SCG	2.3	27	34 ± 5	12.6	41 (0.5)	6	Intense anterior chamber inflammation (24.5); tube occlusion (5.2); endophthalmitis (3.5).
Daniel et al., 2019^(14)^	BGI MGI AGV	PCG and SCG	2	60	23.6 ± 5.8	17.6 ± 6.3	93 (1)	48	Tube exposure (13); endophthalmitis (8); hypotony (8)
Arad et al., 2019^(34)^	XEN-augmented BGI	PCG and SCG	6.5	10	32	18.9	60 (1)	12.7	Surgical revision** (30)
Durai et al., 2021^(35)^	AADI TREC	Aniridia	15.7 ± 9.5 11.7 ± 12.3	18 12	32.5 ± 11.4 27.2 ± 10.9	14.1 ± 2.8 9.6 ± 6.6	83 17	12	retinal detachment (8.3% TREC), cataract (41.7 TREC); shallow anterior chamber (5.6 AADI)
Promelle et al., 2021^(36)^	AGV w/ MMC	PCG and SCG	6.4 ± 5.1	81	30.4 ± 8.1	19.9 ± 6.8	72 (1) 58 (2) 35 (5)	96	Hypotony (30.8); flat anterior chamber (14.8); cataract (11); corneal opacity (15); choroidal detachment (5)
Khan et al., 2021^(37)^	AAID AGV	PCG and SCG	7.8 ± 5.4 9.0 ± 5.2	56 70	32.2 ± 8.2 33.1 ± 7.9	~16.7 ~18.2	69.9 (2) 66.8 (2)	13.8 25.3	Choroidal detachment (12.5/1.4); Retinal detachment (5.4/4.3); Endophthalmitis (0/2.9); Encysted bleb (0/8.6); Cataract (0/4.3); Hypotony (3.6/1.4)
Jacobson et al., 2022^(38)^	BGI	PCG and SCG	7.7 ± 5.7	106	26 ± 9	16 ± 6	64	57.6	Retinal detachment (6.6), hypotony (3.8); suprachoroidal hemorrhage (0.9);
Jacobson et al., 2022 ^(39)^	BGI AGV	CEU	5.4 ± 7.0	7	32.9 ± 11.0	12.7 ± 2.2	57	72	N/A

AGV= Ahmed glaucoma valve; yrs.= years; #= number; IOP= intraocular pressure; mos.= months; AGVs= Ahmed glaucoma valve of silicone; AGVp= Ahmed glaucoma valve of polypropylene; w/ MMC= with mitomycin-C; w/o MMC= without mitomycin-C; BGI= Baerveldt glaucoma implant; AADI= Aurolab aqueous drainage implant; MGI= Molteno glaucoma implant; GFCS= glaucoma following cataract surgery; PCG= primary congenital glaucoma; SCG= secondary congenital glaucoma; CEU: congenital ectropion uvea. * Data is presented as mean or mean ± standard deviation; ** Authors did not present specific percentage values for each complication; NA= not available.

### Types of GDD

Various types of GDDs have been used in children, presenting varied materials and sizes, with or without a flow restriction system. In this review, we reviewed the outcomes of the following GDDs implanted in children: AGV, Molteno glaucoma implant (MGI), Baerveldt glaucoma implant (BGI), and Aurolab aqueous drainage implant (AADI).

Table 1 shows data pertaining to age at the time of GDD implantation, number of eyes operated, initial and final IOP, success rate, follow-up (months), complications, and comparisons among GDDs reported by various authors.

## VALVED GDD

The main advantage of valved GDD is its safety since the unidirectional outflow of aqueous humor prevents potential blinding complications compared to the nonvalved devices^(21,22,23,26,29,30,31,40,41,42,43,44,45,46,47,48)^. Nevertheless, these devices presented earlier encapsulation, resulting in lower long-term success rates compared to the nonvalved GDD(24,37).

Krupin eye valve (KEV) was the first valved glaucoma implant, described in 1980. Its surface area is 184 mm^2^ and it has been less frequently used than the AGV^(49)^. It was not included in this review as we could not retrieve any study on KEV in pediatric glaucoma.

The AGV was approved for clinical use in 1993. It is commonly used in adults and children and produced in two types of materials: polypropylene and silicone. Originally, these GDDs had a restrictive mechanism designed to allow an unidirectional flow, avoiding hypotony^(21,22,23,26,29,30,31,40,41,42,43,44,45,46,47,48)^. A comparison between these two materials showed longer IOP control in the silicone group compared with the polypropylene group after two years of follow-up^(23)^. Therefore, the silicone model has been preferred. In addition, two models of AGV are available which differ in their surface area: FP7 (mostly used in adults; surface area: 184 mm^2^) and FP8 (generally used in children; surface area: 96 mm^2^)^(23)^.

### Nonvalved GDD

The nonvalved GDDs have no unidirectional restrictive mechanism to prevent the retrograde flow of aqueous humor. The last silicone version of MGI (MOLTENO 3) has two size models (185 and 245 mm^2^)^(19)^. BGI has been the main nonvalved device investigated in GDD studies in pediatric glaucoma^(20,24,34,25,50,51)^. This silicone implant is currently available in two sizes: 250 mm^2^ and 350 mm^2^.

AADI was first introduced in India and is a nonvalved, low-cost device derived from BGI, presenting a 350 mm^2^ plate area^(28,33,52)^. Susanna GDD (SGDD) is another nonvalved flexible silicone GDD with a length of 17.4 mm and a width of 14.3 mm^(53)^. It was not included in this review because no study has evaluated SGDD in pediatric glaucoma in the study reference period^(54)^. Table 1 summarizes relevant data from studies evaluating the use of GDDs in various types of pediatric glaucoma. One study has also compared the same valved GDD made of two different materials^(23)^. Other studies have compared valved GDD and nonvalved GDD^(24,37,27)^; two nonvalved GDD^(33,27)^ and two nonvalved versus one-valved GDD^(14)^. Lastly, some studies have compared the same valved GDD with and without intraoperative MMC^(22)^ and evaluated an XEN-augmented nonvalved GDD implantation^(34)^. One study compared a nonvalved GDD and TRAB in secondary glaucoma^(35)^.

### Surgical technique

In children, most GDDs have been implanted in the temporal superior scleral quadrant. The fornix-based approach using a conjunctival incision is more frequently used^(13,14,30)^, but the limbic-based flap has also been described^(16,31)^. The anterior chamber has been predominantly chosen as the site of tube placement^(19)^. However, *pars plana* placement has been adopted in selected eyes with secondary glaucoma such as aniridia, Axenfeld-Rieger syndrome, and Peters’ anomaly^(50,51,55)^. In these cases, the anterior chamber angle is often compromised due to the presence of extensive peripheral anterior synechiae, hampering the tube placement in the anterior chamber through a limbal approach. In cases of corneal opacification, combined endoscopic vitrectomy with posterior placement of GDD can be employed, but with poor success rates^(56)^.

The advantages of the *pars plana* approach include a low risk of corneal contact and less local scleral erosion compared to the limbal insertion^(51)^. There is no consensus on the use of MMC to prevent bleb encapsulation in GDD implants, and most studies have shown no advantage conferred by MMC^(40,22)^. One study in adults investigated the use of a new technique that entails the application of a thin piece of cotton soaked with MMC over the AGV plate; the results showed significantly lower IOP and higher success rates compared with conventional application^(40)^. A recent study using AGV with adjunctive MMC in childhood glaucoma showed controlled IOP in 35% of children at 5-year follow-up^(36)^.

Supra-tenon capsule placement is another approach to prevent plate encapsulation^(45,57)^.

An *ab-interno* approach with AADI was found to be safe and resulted in significant IOP reduction with low endothelial damage^(58)^.

### Indications

In most studies, GDD was used after failure of the primary surgical approach (goniotomy or trabeculotomy). However, GDDs were employed as the first-line treatment in some cases in which angle surgeries have a high risk of failure, such as phakomatosis^(59)^, aphakic glaucoma^(60,61)^ and aniridia^(35,62)^. Chen et al.^(13)^ evaluated AGV implanted as the first surgical approach in patients with PCG or JOAG (age < 18 years), uveitic glaucoma, or secondary glaucoma. Their results are summarized in Table 1.

Pakravan et al. evaluated 95 eyes with aphakic and PCG in whom AGV (FP7) was implanted after a lack of response to medical and other surgical interventions, except patients who had less than six months of follow-up and prior cyclodestructive surgeries. The mean (± standard deviation) age at the time of AGV implantation was 94.7 ± 77.8 months (range: 4-276)^(16)^.

Al-Haddad et al. evaluated the outcomes of AGV (FP7) implantation in 11 eyes with pediatric glaucoma associated with scarring due to at least two prior surgeries^(30)^, while El Gendy^(24)^ compared single-stage BGI (350 mm^2^) and AGV (S-2) implants in 31 eyes with congenital or secondary glaucoma. An important advantage of GDD over other surgical approaches in children is the need for fewer examinations under general anesthesia^(14)^.

AADI and AGV showed a similar reduction in IOP at the final follow-up in refractory pediatric glaucoma; however, the AADI group was associated with lesser use of antiglaucoma drugs and a lower rate of subsequent surgeries^(37)^.

Other recent studies investigated the results of GDD implants in different forms of secondary glaucoma^(38,39,56,62)^. In aniridia, glaucoma developed in 52% of eyes, half of which required IOP-lowering surgery. GDD implants were the main choice of procedure and presented the best success rate (71%) (mean follow-up period: 14.2 ± 15.4 years). This study showed a better success rate of BGI (74%) compared to AGV (63%), but the latter presented less tube revision^(62)^.

Eyes with congenital ectropion uvea may require multiple glaucoma surgeries and only a GDD implant might not be sufficient to control the IOP^(39)^.

### Clinical outcomes

#### IOP control

The definition of success varied among studies due to several factors, such as GDD type, use of MMC, number of previous surgeries, medications, and type of glaucoma^(13,15,19,22,30,31,34,40,50,62)^. Regarding the number of medications, most studies showed a significant reduction in the amount of medication after the use of any GDD implant^(13,21,22,25,26,29,31,33)^. The main criteria for defining success in the studies included in this review were IOP reduction to <21 mmHg-with or without medications-as well as a >20% decrease from the initial IOP level^(16,20)^. In summary, in 19 studies, the mean IOP levels measured at final follow-up visits were <18 mmHg, with success rates varying from 6% to 97% (Table 1). Complete success was defined in most studies as the reduction in final IOP without any medications. However, the definition of qualified success varied among the studies, and the critical details are presented individually. The final IOP was the average of all measurements at the last follow-up.

Chen et al. showed IOP reduction from 29.2 ± 9.7 mmHg preoperatively to 15.2 ± 3.5 mmHg up to 10 years after the AGV implant. Mean IOP reduction was 13.0 mmHg (95% confidence interval: 8.8-17.3) at the final follow-up. The success rate calculated at 5-year follow-up was 55.0% with no significant difference among types of glaucoma and in the number of medications. The median survival time was 6.8 years for all eyes (4.2 years for those with silicone GDD and 10.5 years for those with polypropylene GDD). Nevertheless, only the implant model was a risk factor associated with surgical failure, with eyes that underwent a polypropylene GDD implantation showing a lower risk ratio (0.45)^(13)^.

Mandalos et al.^(17)^ categorized the success criteria as either qualified or complete success. At the last postoperative visit, the mean IOP was 15.5 ± 8.4 mmHg on a median of 0 (range: 0-3) medications. Both IOP and the number of medications were significantly lower than baseline. In the last postoperative visit, 46 eyes (66.7%) were deemed successful, and complete success was achieved in 34 eyes (49.3%). Twenty-three eyes (33.3%) were considered failures^(17)^.

Pakravan et al.^(16)^ showed a cumulative probability of success of 90% in patients with PCG and aphakic glaucoma who underwent AGV implantation during the first year of life; however, the survival rate decreased to 52.5% in PCG and 71.5% in aphakic patients at the five-year follow-up visit. The mean IOP before surgery was 31.5 ± 8.0 and 28.9 ± 6.1 mmHg, respectively, which reduced to 17.7 ± 6.3 and 16.0 ± 5.9 mmHg at year 3, respectively. The mean postoperative IOP level did not differ between patients with PCG and those with aphakic glaucoma.

Other authors^(21,22)^ evaluated AGV implantation during the first two years of life and showed cumulative survival rates of 73.8% after 12 months and 63.3% after 24 months. However, 85.8% of eyes required antiglaucoma medication for IOP control; notwithstanding the number of medications was lower after 24 months compared to preoperative level (mean: 1.45 ± 1.25 versus 2.67 ± 0.87, respectively).

Low-cost GDD has also shown good clinical results. In a prospective interventional study, implantation of AADI was found to be safe and effective in reducing IOP levels compared to AGV and BGI^(28,33)^. A recent study showed similar IOP reduction with AADI and AGV, but fewer glaucoma medications and reoperations when AADI was employed^(37)^.

A few studies have compared the outcomes of valved and nonvalved implants (Table 1). One study showed significantly higher postoperative IOP in the AGV group compared to BGI (24 mmHg versus 17.4 mmHg; p=0.03), with no difference in the postoperative medications^(24)^. Senthil et al. compared AGV with low-cost nonvalved GDD (AADI) and found a significantly lower mean IOP and significantly fewer glaucoma medications in the latter group^(52)^.

A few studies have compared the use of MMC to prevent plate scarring. Mokbel et al.^(43)^ showed that the use of intraoperative MMC in eyes that have undergone excision of the capsule around failed AGV implantation did not increase the final success rate. Al-Mobarak et al. compared the effect of intraoperative use of MMC in AGV implants. They reported longer mean survival time in children who were not treated with MMC, as well as fewer postoperative glaucoma medications^(22)^. A recent study showed a complete success rate of 35% with AGV implant with adjunctive MMC at 5-year follow-up^(36)^.

Aiming the prevention of plate encapsulation, Elhefney et al. evaluated a supra-Tenon’s capsule implantation of AGV. This technique showed a success rate of 81.9%, with few postoperative complications in children with refractory glaucoma^(45)^. Excision of the capsule plate resulted in short-term resolution and IOP control, but there are no proven long-term benefits of this procedure^(44,46)^.

One study identified Hispanic ethnicity and female sex as risk factors for failure after a GDD implant^(48)^.

The implantation of a second GDD improved the survival rates^(41)^, although it presented a higher risk of failure and a higher incidence of complications, such as decreased vision and corneal decompensation^(13,48)^. The angular surgical approach is an interesting option in case of failure of the GDD implant. A recent prospective study compared the outcomes of AGV revision with those of visco-trabeculotomy in children with primary and secondary glaucoma who showed failure of AGV implant. At the 12-month follow-up, eyes that underwent visco-trabeculotomy showed significantly better success rates compared to the AGV revision group^(46)^.

#### Complications

Compared to adults, pediatric patients showed different frequency of complications associated with GDD. Most studies presented either a variable rate of GDD complications or did not describe them. One of the most common GDD-related complications in the late postoperative period was bleb encapsulation^(27,63)^. Even so, severe complications, such as endophthalmitis, may occur in children and have been reported in most studies (Table 1). Hypotony was more frequently observed after implantation of nonvalved GDD since valved implants restrict the flow of aqueous humor at lower IOP levels^(52,27)^. Nevertheless, a few studies have shown cases of hypotony after implantation of a valved GDD^(41)^. The incidence was variable, with some studies having incidence rates as high as 40%^(27)^. To prevent hypotony, some additional approaches can be employed, such as AGV ligature, which showed both less hypotony and choroidal effusion^(41)^. Cataract was more commonly observed in nonvalved GDD implants and was mostly associated with severe hypotony episodes^(41)^. A significant rate of cataract progression (22.9% of phakic patients) in children has also been observed^(27)^; however, it was not clear whether it was associated with GDD.

Strabismus is another common complication of GDD implantation in children^(64,65,66)^. A recent study showed more severe limitations and motility disturbances with the use of AGV^(65)^. Exotropia and vertical strabismus were the most frequently observed conditions, which were attributable to both the mass effect and bleb scar that limits eye movements^(65)^. In the study by Vinod et al., *pars plana* insertion of a GDD was not associated with an increased risk of strabismus compared to anterior chamber insertion; however, the small sample size and retrospective study design were limitations of the study^(51)^. Furthermore, the inability to remove the hyaloid during pediatric vitrectomy may contribute to subsequent *pars plana* tube obstruction^(25,51)^. Vitreous occlusion may occur even 9 years after implantation, with an average of 3 years after *pars plana* BGI implant^(51)^. Anterior dislocation and rotation of the proximal tube tip were more commonly observed in children than adults. Some authors attributed this to the relatively lower scleral rigidity in children^(31,40,51)^. Moreover, there were some cases of tube movement causing corneal edema and cataract^(42)^.

Kalinina et al. evaluated corneal endothelial cell density (ECD) after AGV implantation in children with uveitic glaucoma and showed a decrease in ECD after AGV implant compared to eyes not submitted to that surgery^(42)^. A recent approach, called XEN-augmented Baerveldt implantation, was developed to prevent corneal touch; it consists of an XEN stent connected to the distal end of the Baerveldt tube. In children, this adapted GDD showed no signs of corneal decompensation in the short-term, but has the disadvantage of using two implants^(34)^. In a recent study, 4.7% of children showed the need for corneal grafting after GDD implantation^(67)^.

Tube exposure is also an important complication that can lead to catastrophic results and may occur at any time after surgery in patients with pediatric glaucoma and uveitis^(16,25,29)^. The reported frequency of this complication ranges from 2.4% to 12% (Table 1). Tube revision was the most common surgical intervention after GDD implantation (21.8%) and tube exposure was the most frequent reason for the revision. Other complications associated with tube revision included cataract and strabismus; however, no cases of endophthalmitis were reported^(13)^.

Suprachoroidal hemorrhage was an unusual complication reported, as well as retinal detachment and choroidal effusion^(23)^. Nevertheless, the occurrence of suprachoroidal hemorrhage is rare even with the use of nonvalved GDD^(68)^.

In addition, Senthil et al., compared nonvalved (AADI) with valved (AGV) GDD, and showed a slightly higher incidence of sight-threatening complications in the AADI group^(52)^.

## DISCUSSION

Due to methodological and ethical issues, most published studies evaluating outcomes of GDD in children were retrospective. In addition, the use of GDD has been considered as one of the secondary surgical treatment alternatives. Only one randomized controlled clinical trial comparing BGI implant versus TRAB was retrieved, but it was discontinued due to complications, including retinal detachment and tube extrusion^(69)^. Few studies have employed GDD as the first surgical approach for secondary glaucoma^(31,62)^.

Nassiri et al. published a review specifically focused on the use of AGV in children^(15)^. However, in the present study, we reviewed the outcomes of all commercially available GDDs used in pediatric glaucoma, including studies comparing the outcomes of GDDs versus other surgical procedures.

Currently, there is no clear consensus on the optimal surgical strategy after the failure of angle surgery in childhood glaucoma. GDD implantation has been commonly used in refractory pediatric patients, but most eyes that underwent GDD surgery required additional ancillary treatment, mainly topical medications. Pediatric GDD surgery, albeit tricky, is overall safe, effective, and can have satisfactory medium to long-term outcomes.

The reported success rates for GDD implantation in refractory PCG vary between 16.4% and 97%^(16,26)^. Despite variability in the success rates, AGV implantation has shown better outcomes in aphakic glaucoma compared to that in refractory PCG^(45)^. The success of a glaucoma implant surgery in lowering IOP largely depends on the flow of aqueous humor through the GDD drainage system and at the surgical site. The thickness of the bleb wall determines the resistance to aqueous flow, and Tenon’s capsule plays a major role in determining the bleb wall thickness^(45)^. The supra-Tenon’s capsule implantation technique, using AGV^(45)^, showed good but similar success rates when compared to the subcapsular approach. Nevertheless, thinner capsular wall was associated with significantly better outcomes of GDD implantation as demonstrated by anteriorsegment OCT^(70)^. Furthermore, there is no consensus on the intraoperative application of MMC, but most studies have shown that it confers no advantage. Moreover, the application of MMC after the excision of the capsule around failed AGV implants did not increase the success rates^(44)^. Nevertheless, a recent noncomparative study showed a 35% success rate with the use of MMC at 5-year follow-up^(36)^.

There is a paucity of data regarding the hypertensive phase in childhood glaucoma. The hypertensive phase in children seems to be compared to adults, and it has been observed more commonly in refractory PCG compared to postlensectomy eyes^(16)^. On comparing the singlestage (tube insertion during the primary surgery) and the two-stage implantation (tube insertion performed days after the fixation of the plate) approaches, there was no significant difference in the probability of success (71% versus 85%, respectively; p=0.32), even after considering the location of the tube insertion (anterior chamber, 71%; posterior chamber, 79%).

Variable success rates after AGV implantation may be associated with differences regarding the surgical history of children. Most PCG patients had undergone glaucoma surgery before AGV implantation; however, AGV implant was the first choice for a specific group of children, particularly the aphakic glaucoma group^(16)^. Although implantation of AGV in older children was associated with higher success rates, even in uveitic glaucoma^(29,43)^, the long-term outcomes of AGV implantation in pediatric patients with uveitis are not well characterized. AGV may be a good alternative in the management of elevated IOP in this population, but glaucoma medications are frequently required postoperatively^(29)^. In pediatric glaucoma following cataract surgery, one study showed good long-term outcomes with AGV and BGI^(32)^.

Age at implantation (longer survival rate at an older age) and the GDD model were found to influence success rates. Success rates after implantation of one GDD ranged from 63% to 93% after 1 year and 30% to 70% after 5 years. Age at the time of shunt implantation was associated with the likelihood of failure, with younger eyes showing lower success rates. The severity of glaucoma at presentation is inversely correlated with the surgical success rate in children^(17)^. We speculate that lower age might be a risk factor for failure because patients diagnosed at younger age may have poorly developed outflow pathway, more intense wound healing process, frequent eye rubbing, and difficulties in maintaining a medication regimen.

Clinical comparison between adult and pediatric GDD implantation is difficult, because of several biomechanical and anatomical differences. Although GDD failed earlier in adults than in children, there was no significant difference between adults and children regarding IOP control. Moreover, IOP levels and the number of topical medications used were similar throughout all postoperative visits^(19)^.

A relatively high complication rate should be expected, but in most cases, these can be managed successfully^(17)^. Tube-related complications may be observed in up to one-third of operated eyes, with both the valved implants and modern surgical techniques being associated with scant improvement in children. For instance, eyes that underwent the Baerveldt single-stage surgery showed a higher risk of complications, while fewer complications were observed using an absorbable suture ligation^(24)^.

Altogether, studies investigating the use of GDD in children show considerable heterogeneity regarding the methodology and data quality. Notwithstanding the risks of generalization about the best shunt device and surgical approach, there are variable, but good IOP results in the medium-term follow-up of treating pediatric glaucoma with GDD.
